# Butyrate and Lauric acid effect on Astrocytes and Neurons in Alzheimer’s disease

**DOI:** 10.1002/alz.092277

**Published:** 2025-01-03

**Authors:** R.M. Uththara Sachinthanie Senarath, Lotta E Oikari, Prashant Bharadwaj, Vijay Jayasena, Ralph N Martins, Warnakulasuriya Mary Ann Dipika Binosha Fernando

**Affiliations:** ^1^ Edith Cowan University, Perth, Western Australia Australia; ^2^ Centre of Excellence for Alzheimer’s Disease Research and Care, Nedlands, Western Australia Australia; ^3^ QIMR Berghofer Medical Research Institute, Herston, QLD Australia; ^4^ Australian Alzheimer’s Research Foundation, Perth, Western Australia Australia; ^5^ Western Sydney University, Sydney, NSW Australia; ^6^ Macquarie University, Sydney, NSW Australia; ^7^ Centre of Excellence for Alzheimer’s Disease Research and Care, School of Medical and Health Sciences, Edith Cowan University, Joondalup, Western Australia Australia; ^8^ Australian Alzheimer's Research Foundation, Perth, Western Australia Australia

## Abstract

**Background:**

Recently, there has been substantial interest in investigating the role of short‐chain fatty acids (SCFAs) and, medium chain fatty acids (MCFA) in the neuroinflammation associated with Alzheimer’s disease (AD). Specifically, butyrate (SCFA) and lauric acid (MCFA) have demonstrated potential in alleviating neuroinflammation and reducing toxicity associated with amyloid proteins. Additionally, they have been found to enhance mitochondrial function and reduce neuronal hyperactivity. However, the mechanisms by which butyrate and lauric acid either suppress or activate astrocytes and neurons, as well as how they influence Aβ‐mediated activation of astrocytes and neurons, remain unclear. This study aims to address this knowledge gap by investigating the independent and synergistic effects of butyrate and lauric acid on AD pathogenesis—a research endeavour not undertaken previously.

**Method:**

Butyrate and lauric acid were administered independently and synergistically to cultures of astrocytes, neurons derived from induced pluripotent stem cells (iPSCs) obtained from healthy human control subjects. Inflammatory responses of astrocytes and neurons were measured under synthetic amyloid beta stimulating conditions similar to AD. Legend Plex Human Inflammatory Panel was used to quantify the response of inflammatory cytokines (Interleukin IL‐1β, IL‐6, IL‐8, IL‐18, IL‐33, etc.) to inflammatory stimuli. The statistical analysis was carried out using the GraphPad Prism version 10.1.2. One‐way ANOVA was used to analyse the data.

**Result:**

Butyrate and lauric acid, independently and synergistically demonstrate a positive association with pro‐inflammatory cytokines. There were negative association with anti‐inflammatory cytokines in neuron, astrocytes and spontaneous co‐cultures of astrocytes and neurons.

**Conclusion:**

The findings indicate that both butyrate and lauric acid inhibit inflammation in Alzheimer’s‐induced astrocytes and neurons. Ongoing investigations aim to evaluate their effects on various cytokines and determine the responsible genes through qPCR analysis.

